# Acute appendicitis and ulcerative colitis: a population-based sibling comparison study

**DOI:** 10.1136/bmjgast-2022-001041

**Published:** 2022-11-29

**Authors:** Miguel Garcia-Argibay, Ayako Hiyoshi, Scott Montgomery

**Affiliations:** 1 Clinical Epidemiology and Biostatistics, School of Medical Sciences, Faculty of Medicine and Health, Örebro universitet, Örebro, Sweden; 2 Department of Public Health Sciences, Stockholm University, Stockholm, Sweden; 3 Department of Epidemiology and Public Health, University College London, London, UK; 4 Public Health, Department of Social Medicine, Osaka University Graduate School of Medicine, Osaka, Japan; 5 Clinical Epidemiology Division, Department of Medicine, Karolinska Institutet, Solna, Sweden

**Keywords:** ulcerative colitis, appendicitis, inflammatory bowel disease, genetics

## Abstract

**Objective:**

To assess the inverse relationship between acute appendicitis and ulcerative colitis (UC) using a sibling comparison design to adjust for unmeasured familial genetic and environmental factors.

**Design:**

The cohort comprised 3.1 million individuals resident in Sweden between 1984 and 2018 with the linkage of several Swedish national registers. Fitting Cox hazards models, we calculated the risk for developing UC in individuals with and without acute appendicitis by the age 20 years adjusting for several potential confounding factors. Further, we performed sibling-stratified analyses to adjust for shared unmeasured familial confounding factors.

**Results:**

During 57.7 million person-years of follow-up, 20 848/3 125 232 developed UC among those without appendicitis (3.63 (3.59–3.68) per 10 000 person-years), whereas only 59/35 848 people developed UC among those with appendicitis before age 20 years (1.66 (1.28–2.14) per 10 000 person-years). We found a decreased risk for developing UC in those with acute appendicitis by the age 20 years compared with individuals who did not have appendicitis by this age (HR=0.37 (95% CI 0.29 to 0.48)). When adjusting for shared familial confounders, we observed only a slight attenuation in this association (HR=0.46 (95% CI 0.32 to 0.66)).

**Conclusion:**

Individuals who had acute appendicitis by late adolescence showed a decreased risk for developing UC compared with those who did not. Genetic and shared familial environmental factors seem to potentially play only a small role in this relationship. Our results suggest an independent association of acute appendicitis, or its underlying causes, with UC risk.

**WHAT IS ALREADY KNOWN ON THIS TOPIC**

- Previous research showed an inverse relationship between appendicitis and ulcerative colitis (UC).
- Recent evidence suggested that this relationship seems to be explained by genetic or environmental factors.

**WHAT THIS STUDY ADDS**

- This study showed that genetic and environmental factors shared by full siblings explain only a small part of the association of acute appendicitis with UC risk.
- A direct relationship between early appendicitis, or its causes, and UC seems to explain the reported reduced risk of UC.

**HOW THIS STUDY MIGHT AFFECT RESEARCH, PRACTICE OR POLICY**

- This study provides convincing evidence that the association of appendicitis with UC is not due to environmental or genetic confounding.

## Introduction

Ulcerative colitis (UC) is an idiopathic, chronic, relapsing inflammatory bowel disease (IBD) characterised by mucosal inflammation of the colon and rectum. The prevalence of UC has been increasing worldwide^1^ with an estimated prevalence of 7.6–246.0 diagnoses per 100 000.^2^ Although the aetiology of UC is unclear, research has shown that it is a multifactorial disease, involving both genetic and environmental factors.^3–5^ In recent decades, the discovery of genetic factors associated with UC, in particular, the role of the human leucocyte antigen genes, has increased our understanding of the pathogenesis of UC.^6 7^ A recent Genome-Wide Association Study of almost 60 000 individuals (25 305 with IBD) has identified 240 risk loci associated with IBD,^8^ but the genetic contribution of these loci to IBD risk is around 20%,^9^ indicating that environmental variables may play a bigger role in the aetiology of UC. Several studies have investigated environmental risk factors that might be linked to the development of UC.^10 11^ Accumulating evidence indicates that being an active smoker and having a history of appendectomy early in life confer a reduced risk of developing UC.^12–15^ Although these relationships are incompletely understood, the protective association with appendicectomy appears to be due to appendicitis rather than appendicectomy per se,^16 17^ and largely limited to individuals with appendicitis before age 20 years.^17^ One study reported that appendicitis in a relative is also associated with reduced UC risk,^18^ suggesting the association may be due to confounding by familial genetic or environmental factors.

To the best of our knowledge, no study to date has been performed adjusting for unmeasured familial genetic and environmental risk factors shared by family members using sibling comparison methods. In this study, using Swedish general population registers, we sought to assess the relationship between acute appendicitis by late adolescence and UC using a sibling comparison design to adjust for unmeasured familial factors.

## Methods

### Study design and population

This study was based on the linkage of several Swedish registers using the unique personal identification number issued at birth or immigration to all residents in Sweden, namely, the Total Population Register (TPR), the National Patient Register (NPR), the Multigeneration Register, the Longitudinal Integrated Database for Health Insurance and Labour Market Studies (Swedish acronym, LISA) and the Population and Housing Census (Swedish acronym, FOB). The NPR includes inpatient and specialist outpatient care visits since 1964 and 2001, respectively, using the International Classification of Diseases (ICD-7 1964–8, ICD-8 1969–86; ICD-9 1987–96; ICD-10 1997–onward).^19^ The NPR had almost full coverage of inpatient care since 1973, full national coverage from 1987 for inpatients, and full coverage for outpatient services since 2001. We identified all individuals born in Sweden between 1964 and 1997 and living in Sweden between 1984 and 2018 from the TPR, excluding those who died, emigrated or had a diagnosis of UC before the start of follow-up or with unidentified biological parents. Each individual was linked with their full siblings using the Multigeneration Register. Diagnoses were obtained from the NPR, and social and demographic information was obtained from FOB, the TPR and LISA.

The cohort was followed from age 20 years until the occurrence of UC, Crohn’s disease (CD) (ICD-8: 563, ICD-9: 555A 555B 555C 555X, ICD-10: K50), death, emigration from Sweden or the end of the study period (31 December 2018), whichever occurred first. At the end of follow-up, the youngest individuals were aged 21 years (ie, individuals required to have at least 1 year of follow-up) and the oldest were 54 years.

### Definition of UC

UC was defined using ICD codes in the NPR: ICD-7: 572, ICD-8 563, 569, ICD-9 556 and ICD-10 K51. Individuals with an initial UC diagnosis, but followed by at least two CD diagnoses, were classified as having CD and censored at the first diagnosis. Similarly, those with an initial CD diagnosis that was followed by at least 2 UC diagnoses were classified as having UC and censored at the first diagnosis. Given that for ICD-8 codes we only had access to three-digit codes, differentiation between UC and CD was achieved using subsequent diagnoses in the same patients using ICD-9/10 codes. Individuals with only ICD-8 codes were excluded.

### Definition of acute appendicitis

Individuals with acute appendicitis were identified using ICD-8 codes 540–543, ICD-9: 540, 541–543 and ICD-10: K35–K38, and underwent appendicectomy (Swedish Classification of Operation and Major Procedures codes: JEA00, JEA01, JEA10, JEW96 and JEW97) to increase diagnostic specificity.

### Covariates

We used birth year, sex, region (Norrland, Svealand and Götaland), Household Crowding Index (HCI) and Socio-Economic Index (SEI) as potential confounding factors. The HCI was calculated as the total number of occupants divided by the number of habitable rooms, and SEI was defined based on the occupation of the father of the household (or the mother when this information was missing). Information on HCI and SEI was taken from the FOB from 1960 to 1990 every 10 years. Information for both variables was taken from the year closest to birth (when not available, information was taken from the census up to age 10 years).

### Statistical analysis

Individuals’ characteristics were summarised by median, proportions and IQR. Incidence rates (IRs) of UC with 95% Cs) were calculated fitting a Poisson generalised linear model with a log link function. A Cox proportional hazards model, with age as the underlying time scale,^20^ was performed to investigate the relationship between acute appendicitis and UC. We performed unadjusted analyses, followed by an analysis adjusting for birth year, sex, region, HCI and SEI as covariates in the model. To account for shared unmeasured familial confounders, we performed a sibling-stratified Cox model in a subsample of full siblings (individuals with the same biological parents) and stratification was performed by family identification number. This approach automatically adjusts for shared measured and unmeasured confounding factors between siblings.^21^ In the sibling-stratified models, we only included covariates that vary among siblings such as birth year, sex and county. The proportionality assumption was tested by inspecting the Shoenfeld residuals. If this assumption is violated, analyses were stratified by the variable that did not meet the proportionality assumption. Estimates are presented as HRs) with 95% CIs). Data management and statistical analysis were performed using R V.4.1.0.^22^

### Sensitivity analysis

Previous studies have indicated that being a smoker confers protection against UC, so we reran analyses using a subset of the cohort who underwent military conscription assessments and answered questionnaires including smoking behaviour in late adolescence (n=381 416). First, we further adjusted estimates for smoking behaviour to assess if smoking is a confounding factor. We then performed stratified analyses for smokers and non-smokers to investigate whether the relationship between acute appendicitis and UC differs between them. Lastly, given that full coverage in the NPR was achieved after 1987, we repeated all analyses restricting individuals to be born after 1987.

## Results

The cohort included 3 161 080 individuals (2 433 490 individuals nested within 1 026 355 clusters of at least 2 siblings), of whom 1 533 321 (48.5%) were female and 1 627 759 (51.5%) were male. Among those, 20 907 (0.7%) were diagnosed with UC after the age 20 years, with a median (IQR) age at UC diagnosis of 29.9 years (25.1–36.2). The median follow-up time was 17.62 years and a total follow-up time including 57.7 million person-years between 1984 and 2018. A diagnosis of appendicitis before age 20 years was observed in 35 848 (1.13%) individuals, with a similar prevalence for males and females (1.25% and 1.01%, respectively). See table 1 for a description of the cohort’s demographic characteristics.

**Table 1:** Characteristics of the 1968–1998 birth cohort (N=3 161 080)

Variable	Without appendicitis (N=3 125 232)*	Appendicitis (N=35 848)*	P value†
Median follow-up time (years)	18 (10, 27)	9 (7, 13)	<0.001
Age at the end of follow-up (years)	38 (30, 47)	29 (27, 33)	<0.001
Sex			<0.001
Male	1 607 461 (51%)	20 298 (57%)	
Female	1 517 771 (49%)	15 550 (43%)	
Household Crowding Index	1.00 (0.75, 1.33)	1.00 (0.67, 1.00)	<0.001
Missing	165 389	2752	
Socio-Economic Index			<0.001
Agriculture	89 106 (2.9%)	550 (1.5%)	
Low	1 060 511 (34%)	11 183 (31%)	
Medium	1 165 328 (37%)	13 435 (37%)	
High	487 285 (16%)	5792 (16%)	
Other	174 320 (5.6%)	2468 (6.9%)	
Unknown	148 682 (4.8%)	2420 (6.8%)	
Region			<0.001
Götaland	1 425 557 (46%)	15 697 (44%)	
Norrland	417 773 (13%)	5126 (14%)	
Svealand	1 121 744 (36%)	13 512 (38%)	
Unknown	160 158 (5.1%)	1513 (4.2%)	
Age of appendicectomy (years)	29 (24, 36)‡	15 (12, 18)	
Ulcerative colitis	20 848 (0.7%)	59 (0.2%)	<0.001
Crohn’s disease	12 003 (0.4%)	93 (0.3%)	<0.001

*Median (IQR); n (%).

†Wilcoxon rank sum test; Pearson’s χ^2^ test.

‡Individuals who underwent appendicectomy for reasons other than acute appendicitis.

The IR of UC was 1.66 (1.26–2.14) events per 10 000 person-years in those with acute appendicitis, and 3.63 (3.59–3.68) events per 10 000 person-years among those without appendicitis before age 20. People with acute appendicitis had a 56% decreased risk for developing UC compared with individuals who did not have appendicitis (HR=0.44 (95% CI 0.34 to 0.57). Similar estimates were obtained after adjusting for birth year, sex, region, HCI and SEI (HR=0.37 (95% CI 0.29 to 0.48)). Further, when estimates were adjusted for shared unmeasured familial factors by sibling comparison analysis, the risk slightly increased towards the null (HR=0.46 (95% CI 0.32 to 0.66); see table 2).

**Table 2:** Association of acute appendicitis with ulcerative colitis in the 1968–1998 birth cohort (N=3 161 080)

Comparison	Model	HR (95% CI)	P value
Between-individual	Unadjusted*	0.44 (0.34 to 0.57)	<0.001
	Adjusted†	0.37 (0.29 to 0.48)	<0.001
Within-sibling	Unadjusted*	0.47 (0.33 to 0.67)	<0.001
	Adjusted†	0.46 (0.32 to 0.66)	<0.001

*Unadjusted model

†Model adjusted for birth year, sex, county, HCI and SEI.

HCI, Household Crowding Index; SEI, Socio-Economic Index.

When examining the relationship between acute appendicitis and UC in a subset of the cohort who underwent military conscription assessments (see online supplemental table S1 for the cohort characteristics), we observed a smaller risk of UC in those with acute appendicitis and who smoked (HR=0.19 (95% CI 0.06 to 0.58)) after adjusting for birth year, sex, region, HCI and SEI, compared with those had acute appendicitis but did not smoke (HR=0.51 (95% CI 0.31 to 0.85); table 3). However, this difference in UC risk between smokers and non-smokers with appendicitis was statistically non-significant (*p_interaction_*=0.107). A statistically significant inverse association between acute appendicitis and UC was observed adjusting for all the aforementioned potential confounding factors and smoking behaviour (HR=0.40 (95% CI 0.24 to 0.65)). When restricting analyses to those born after 1987 (see online supplemental table S2), results remained largely unchanged (online supplemental table S3).

10.1136/bmjgast-2022-001041.supp1 Supplementary data

**Table 3:** Association of acute appendicitis with ulcerative colitis in the military conscript cohort (N=381 416)

Comparison	Model	HR (95% CI)	P value
Overall	Unadjusted*	0.39 (0.25 to 0.63)	<0.001
	Adjusted†	0.40 (0.25 to 0.65)	<0.001
	Adjusted†+smoking	0.40 (0.25 to 0.65)	<0.001
Smokers	Unadjusted*	0.19 (0.06 to 0.58)	0.004
Non-smokers	Unadjusted*	0.51 (0.31 to 0.85)	0.010

*Unadjusted model.

†Model adjusted for birth year, sex, county, HCI and SEI.

HCI, Household Crowding Index; SEI, Socio-Economic Index.

## Discussion

Results from this general population-based study with prospectively recorded data indicate that individuals with acute appendicitis before the age of 20 years had a lower risk of developing UC later in life compared with those without acute appendicitis. Using a within-sibling comparison design to control for unmeasured familial genetic and environmental confounding, we found that acute appendicitis seems to be associated with protection against the later development of UC. This protective effect seen in conventional analysis was only slightly attenuated after adjustment for shared familial characteristics by comparing siblings.

Although the inverse association between appendicitis and UC has previously been reported,^16–18^ to the best of our knowledge, no prior study had taken advantage of sibling comparison methods to assess this relationship. Our study confirms prior evidence on the protective association of early appendicitis with subsequent development of UC and addresses the question of whether this relationship is driven by genetic or environmental factors. In contrast to conclusions from a previous study,^18^ the small reduction in the association observed in the within-sibling comparisons suggests that shared familial genetic or environmental factors do not largely explain the inverse association between appendicitis and UC, suggesting a rather small contribution from unmeasured factors shared by full siblings. This could imply small shared genetic susceptibility, if any, for both acute appendicitis and UC by which individuals with an underlying genetic predisposition for appendicitis may have a decreased risk for developing UC. Importantly, the small magnitude of the attenuation in the estimates after adjusting for familial confounding suggests that there might be a direct association between acute appendicitis, or its causes, and UC.

The biological mechanism linking appendicitis with reduced UC risk remains elusive. Speculation is that the early exposures that predispose for mucosal immune responses leading to increased risk of inflammation in appendicitis, or appendicitis itself, results in an immune profile that reduces UC risk. This may involve an altered host immune response to the gut microbiota, with possible changes in the composition and metabolic activity of gut microbiota. While this is speculation, it is thought that the mucosal immune response to the microbiota is implicated in the aetiology of UC.^23–26^

Among the strengths of the current study are the size of the cohort and the longitudinal design of over four decades with prospectively recorded coverage from inpatient and outpatient care with a 93% positive predictive value for IBD and a 79-90% for UC.^27^ Moreover, by identifying full-sibling relationships, we were able to adjust for shared genetic and environmental factors within sibling groups. Despite several strengths, the results from this study should be interpreted in view of some potential limitations. Although sibling comparisons adjust for unmeasured familial confounding, residual genetic confounding is still possible as sibling analyses can only adjust for approximately 50% of the shared genetic factors. In addition, varying familial factors cannot be accounted for with sibling comparisons and confounding can be amplified by unique factors from each sibling.^28^ Further, when examining the putative protective effect of smoking for UC within the cohort with military conscription assessment data, misclassification is possible given that smoking was measured once at ages 18–20 years. It is possible that some individuals ceased or started smoking during follow-up or that some individuals did not report their smoking behaviour due to social undesirability. This could have potentially underestimated our estimates assuming that there is a causal protective relationship between smoking and UC. However, the results indicated that smoking is not a confounding factor for the association of appendicitis with UC. Due to power constraints when including only individuals with information on smoking habits, it cannot be ruled out that smoking may be an effect modifier of the appendicitis–UC relationship and further research is needed.

## Conclusions

This cohort study was able to tackle potential confounding by familial genetic and environmental characteristics, as well as smoking. The results indicate that appendicitis by late adolescence is associated with protection against developing UC and that shared genetic environmental factors seem to have only a very small role, suggesting a direct effect of appendicitis, or its causes, rather than shared genetic susceptibility.

## Data Availability

The Public Access to Information and Secrecy Act in Sweden prohibits us from making individual level data publicly available. Researchers who are interested in replicating our work can apply for individual level data at Statistics Sweden: www.scb.se/en/services/guidance-for-researchers-and-universities/.
