# Supplementary material for: Acute appendicitis and ulcerative colitis: a population-based sibling comparison study
Source: BMJ Open Gastroenterol. 2022 Nov 29;9(1):e001041. doi: 10.1136/bmjgast-2022-001041 (PMC9710359; doi:10.1136/bmjgast-2022-001041)
Supplement: Supplementary data [file bmjgast-2022-001041supp001.pdf]

Supplementary information

Acute appendicitis and ulcerative colitis: a population-based sibling comparison study

Miguel Garcia-Argibay[1*], Ph.D., Ayako Hiyoshi[1,2,3,4], Ph.D., Scott Montgomery[1,3,5], Ph.D.

Table S1. Characteristics of the subsample with military conscriptions (n=381,416).

**Characteristic**	**Without Appendicitis (N=373,137)**[1]	**Appendicitis (N=8,279)**[1]
Median follow-up time (years)	13.4 (10.5, 16.2)	11.9 (9.5, 14.5)
Age at the end of follow-up (years)	33.4 (30.5, 36.2)	31.9 (29.5, 34.5)
Sex		
Male	362,201 (97%)	8,033 (97%)
Female	10,936 (2.9%)	246 (3.0%)
Household crowding index (HCI),	1.00 (0.67, 1.00)	1.00 (0.67, 1.00)
Missing	26297	512
Socioeconomic index (SEI)		
Agriculture	7,695 (2.1%)	162 (2.0%)
Low	109,141 (29%)	2,416 (29%)
Medium	141,254 (38%)	3,180 (38%)
High	73,129 (20%)	1,624 (20%)
Other	22,441 (6.0%)	481 (5.8%)
Unknown	19,477 (5.2%)	416 (5.0%)
County		
Götaland	170,381 (46%)	3,594 (43%)
Norrland	51,073 (14%)	1,266 (15%)
Svealand	132,609 (36%)	3,076 (37%)
Unknown	19,074 (5.1%)	343 (4.1%)
Age of appendectomy (years)	26 (22, 30)[3]	16 (13, 18)
Ulcerative colitis	2,210 (0.6%)	18 (0.2%)
Crohn's disease	1,114 (0.3%)	21 (0.3%)
Smoker	124,393 (33%)	2,864 (35%)

[1]Median (IQR); n (%)

[2]Wilcoxon rank sum test; Pearson's Chi-squared test

[3]Individuals who underwent appendicectomy for reasons other than acute appendicitis

Table S2. Characteristics of the cohort born after 1987 (n=812,120).

**Characteristic**	**Without Appendicitis (N=790,249)**[1]	**Appendicitis (N=21,871)**[1]
Median follow-up time (years)	7.46 (5.71, 9.13)	7.38 (5.71, 9.04)
Age at the end of follow-up (years)	27.46 (25.71, 29.13)	27.38 (25.71, 29.04)
Sex		
Male	405,980 (51%)	12,511 (57%)
Female	384,269 (49%)	9,360 (43%)
Household crowding index (HCI),	1.00 (0.67, 1.00)	1.00 (0.67, 1.00)
Missing	84,831	1,837
Socioeconomic index (SEI)		
Agriculture	9,510 (1.2%)	264 (1.2%)
Low	108,798 (14%)	3,105 (14%)
Medium	247,970 (31%)	6,997 (32%)
High	291,036 (37%)	8,217 (38%)
Other	77,899 (9.9%)	1,692 (7.7%)
Unknown	55,036 (7.0%)	1,596 (7.3%)
County		
Götaland	354,352 (45%)	9,708 (44%)
Norrland	100,244 (13%)	2,995 (14%)
Svealand	288,572 (37%)	8,341 (38%)
Unknown	47,081 (6.0%)	827 (3.8%)
Age of appendectomy (years)	22.7 (20.7, 25.0)[3]	13.6 (10.6, 17.0)
Ulcerative colitis	2,738 (0.3%)	24 (0.1%)
Crohn's disease	1,821 (0.2%)	41 (0.2%)

[1]Median (IQR); n (%)

[2]Wilcoxon rank sum test; Pearson's Chi-squared test

[3]Individuals who underwent appendicectomy for reasons other than acute appendicitis

Table S3. Association of acute appendicitis with ulcerative colitis in a cohort born after 1987 (N=812,120).

**Comparison**	**Model**	**HR (95% CI)**	***p***
Between-individual	Unadjusted[1]	0.32 (0.21–0.47)	<.001
	Adjusted[2]	0.35 (0.23–0.52)	<.001
Within-sibling	Unadjusted[1]	0.43 (0.22–0.84)	.01
	Adjusted[2]	0.48 (0.24–0.95)	.02

Note.[1]Unadjusted model; [2]Model adjusted for birthyear, sex, county, HCI, and SEI
